# Supplementary material for: REG3A/REG3B promotes acinar to ductal metaplasia through binding to EXTL3 and activating the RAS-RAF-MEK-ERK signaling pathway
Source: Commun Biol. 2021 Jun 7;4:688. doi: 10.1038/s42003-021-02193-z (PMC8184755; doi:10.1038/s42003-021-02193-z)
Supplement: Supplementary file 2 — Supplementary information [file 42003_2021_2193_MOESM2_ESM.pdf]

REG3A/REG3B promotes acinar to ductal metaplasia through binding to EXTL3 and activating the RAS-RAF-MEK-ERK signaling pathway

Huairong Zhang,[1, 2, 3*] Andrea Liliam Gomez Corredor,[2*] Julia Messina-Pacheco[2*], Qing Li,[4] George Zogopoulos,[5] Nancy Kaddour,[6] Yifan Wang,[5] Bing-yin Shi,[3] Alex Gregorieff,[2] Jun-li Liu,[6#] Zu-hua Gao[2#]

1. Department of Endocrinology and Metabolism, Shanghai General Hospital, Shanghai Jiao Tong University School of Medicine, Shanghai 200080, China
2. Department of Pathology, McGill University and the Research Institute of McGill University Health Centre, Montreal, QC, H4A3J1, Canada
3. Department of Endocrinology, The First Affiliated Hospital of Xi'an Jiaotong University, Xi'an, Shaanxi Province, 710061, China
4. Human Oncology and Pathogenesis Program, Memorial Sloan Kettering Cancer Center (MSKCC), New York, NY 10065, USA
5. Department of Surgery, McGill University and the Research Institute of McGill University Health Centre, Montreal, QC, H4A3J1, Canada
6. Department of Medicine, McGill University and the Research Institute of McGill University Health Centre, Montreal, QC, H4A3J1, Canada

*These authors contributed equally

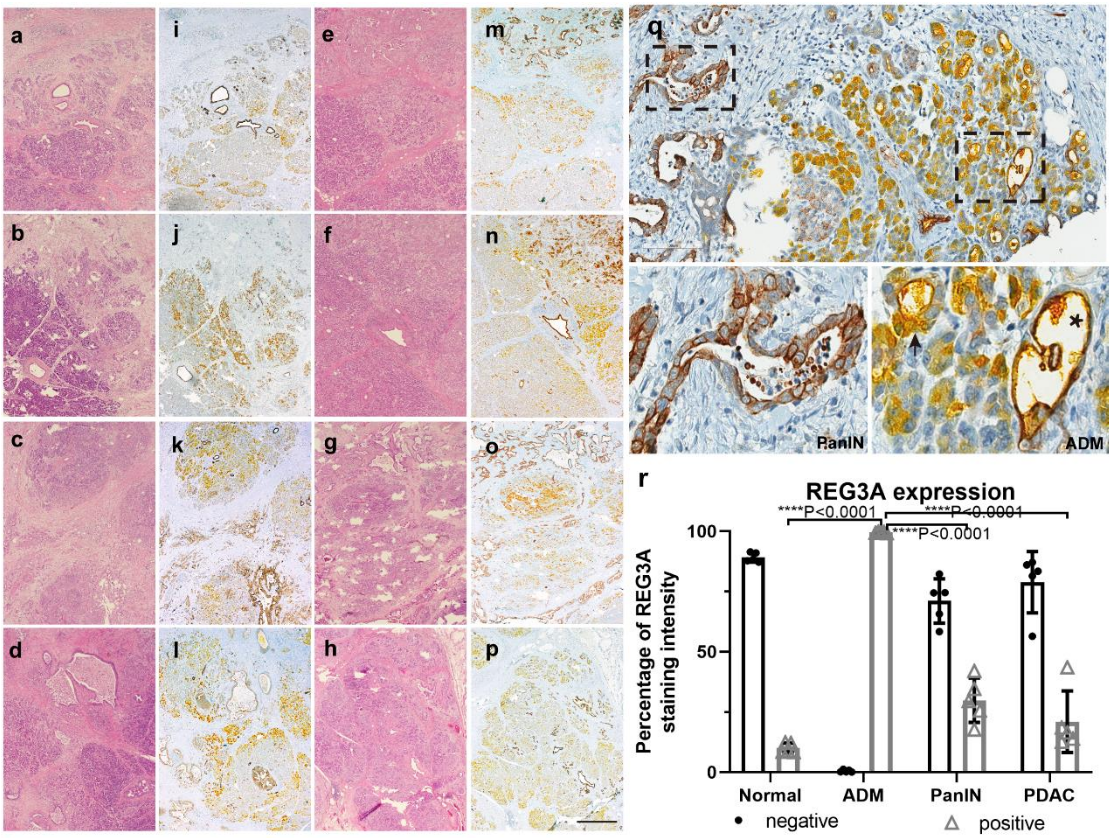

Supplementary Figure 1. REG3A is overexpressed in the ADM area adjacent to human PDAC.

(**a-p**) H&E staining shows histological evidence of transformation from normal acini to ADM (a-d, e-h) with corresponding IHC staining for both REG3A (yellow) and CK19 (brown) (i-l,m-p). Magnification, 40x. scale bar: 2mm. n=9, 8 out of 9 human samples were shown due to limited space. **q** Immunohistochemistry of REG3A (yellow), CK19 (brown) and hematoxylin counterstain on human pancreatic tissue. Representative image with enlarged view showing colocalization of REG3A (yellow) and CK19 (brown) in mature ADM. Nuclei are counterstained in blue. Scale bar: 100μm. Black arrow indicates immature ADM which is predominently REG3A stained. Asterisk indicates relativelly well-developed ADM which is possitively co-stained with REG3A and CK19. **r** Percentage of each phenotypic category (normal vs. ADM vs. PanIN vs. PDAC) displaying negative and positive REG3A staining on human pancreatic tissue (n=9, student's t test).

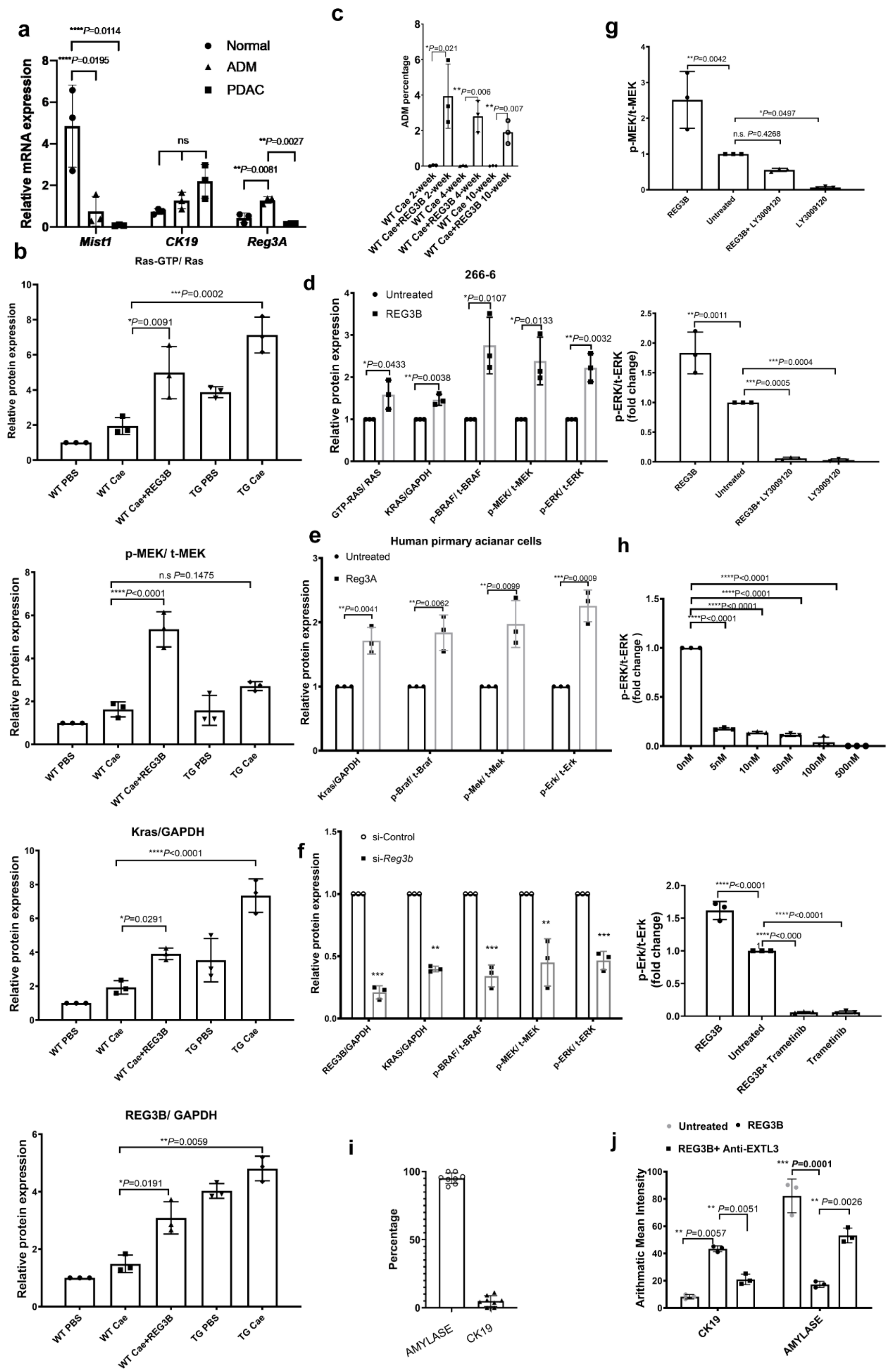

Supplementary Figure 2. Quantification of indicated immunoblots

a RT-qPCR analysis demonstrated increased levels of REG3A, MIST1 and CK19 mRNA in ADM tissue, (n=3, student's t test). **b** Quantifications of the Western blots showing increased p-ERK, p-MEK, KRAS and active RAS in the pancreatic tissue of REG3B-treated WT with caerulein-induced pancreatitis (WT cae) and TG mice with caerulein-induced pancreatitis (TG cae), (n=3, one-way ANOVA). Western blotting of pancreatic lysates shows that TG mice contain 2.7 times more REG3B protein than WT mice. GAPDH serves as a loading control. **c** Quantification of ADM percentage of ADM in 2, 4, 10 weeks after caerulein treatment between REG3B treated and WT mice. N=5, student's t test. **d** and **e** Quantification of western blots demonstrating increased expression of active RAS, p-ERK, KRAS, p-BRAF, p-MEK and p-ERK in the cultured mouse acinar cell line 266-6 (d) and human primary acinar cells (e) after REG3B treatment for 48 hours, (n=3, student's t test). **f** Quantification of western blots showing reduced expression of phosphorylated ERK, MEK, BRAF and KRAS in the 266-6 cell line after *Reg3b* gene knockdown by siRNA. **g** Quantification of western blots showing that LY3009120 blocked REG3B-induced MEK and ERK phosphorylation. (n=3, one-way ANOVA test). **h** Quantification of western blots showing that Trametinib efficiently attenuated ERK phosphorylation in a dose-dependent manner and effectively blocked REG3B-induced ERK phosphorylation. (n=3, one-way ANOVA test). **i** Percentage of AMYLASE-positive and CK19-positive cells in untreated mouse primary acinar cells (n=3, student's t test). **j** Quantification of arithmatic mean intensity of CK19 and AMYLASE in REG3B with or without EXTL3 neutralizing antibody treated mouse primary acinars cells. (n=3, one-way ANOVA test). Data are presented as means ± SD; *P < 0.05; **P < 0.01; ***P < 0.001; n.s, non-significant (P > 0.05).

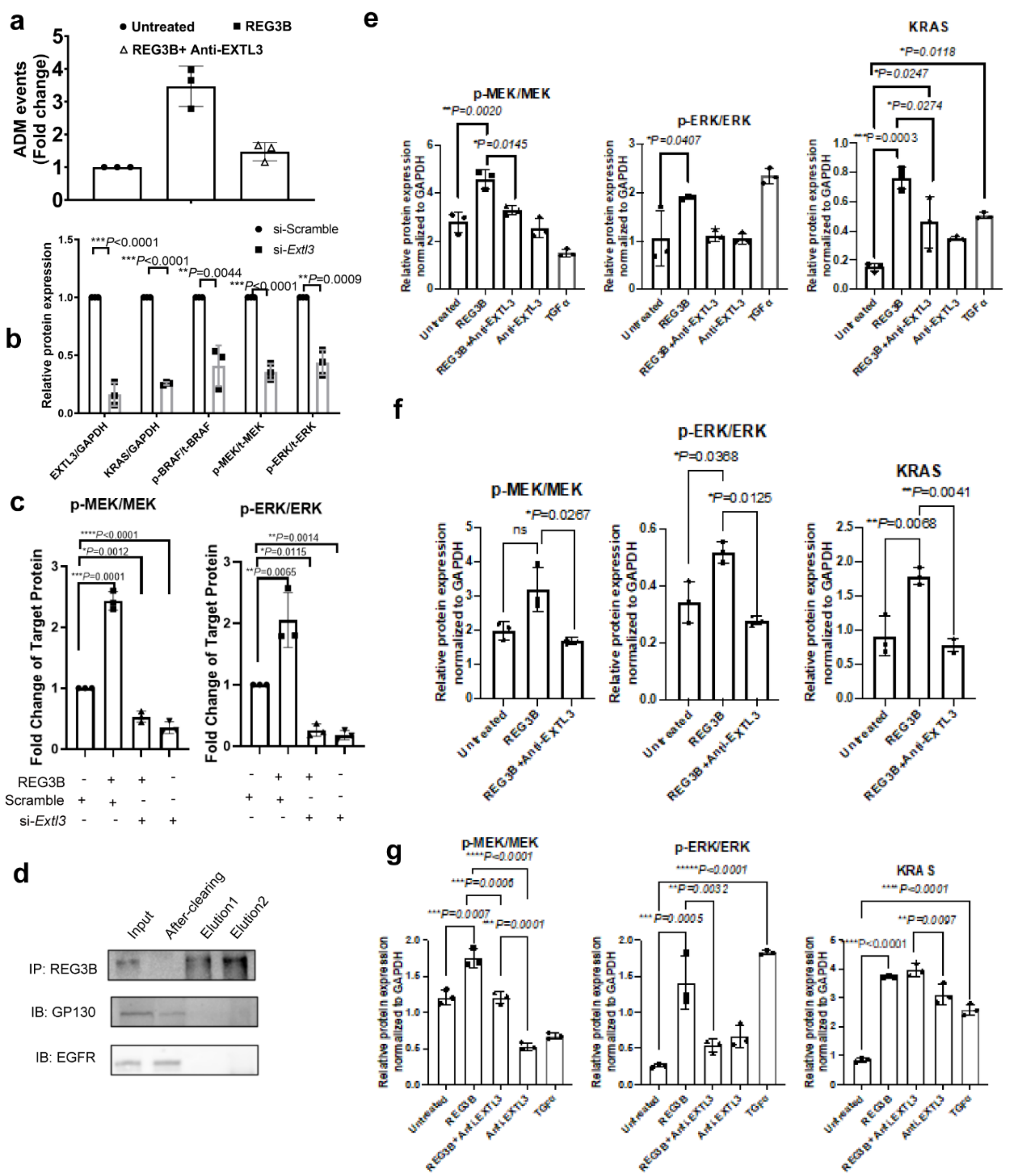

Supplementary Figure 3. Quantification of indicated immunoblots and confocal immunofluorescence microscopy images demonstrating the role of EXTL3 as a receptor for REG3B in ADM

a Mean intensity quantification of confocal immunofluorescence images demonstrating inhibition of REGB-induced ADM, as indicated by increased expression of acinar marker AMYLASE and decreased expression of the ductal marker CK19 upon EXTL3 treatment (one-way ANOVA test). **b** Bar graph showing the decrease in ADM events in 3D culture of mouse primary acinar cells after the 5-day REG3B with EXTL3 neutralizing antibody combination treatment (n=3,15 fields each sample; one-way

ANOVA test). **c** Quantifications of Western blots indicating reduced REG3B. KRAS and phosphorylated BRAF, MEK and ERK upon knockdown of *Extl3* by siRNA (20nM) in 266-6 cell line (n=3, one-way ANOVA test). **d** CO-IP on GP130 and EGFR with REG3B in 266-6 cell line. **e-g** Quantifications of Western blots demonstrating EXTL3 neutralizing antibody-mediated inhibition of REG3B-induced phosphorylation of MEK and ERK and expression of KRAS in (e) AR42J cells and (f) mouse primary acinar cells after treatment for 48 hours and (g) human primary acinar cells after treatment for 30 minutes. (n=3, one way ANOVA test). Data are represented as means ± SD. *P < 0.05, **P < 0.01, ***P < 0.001. Non-significant (n.s.) if P > 0.05.

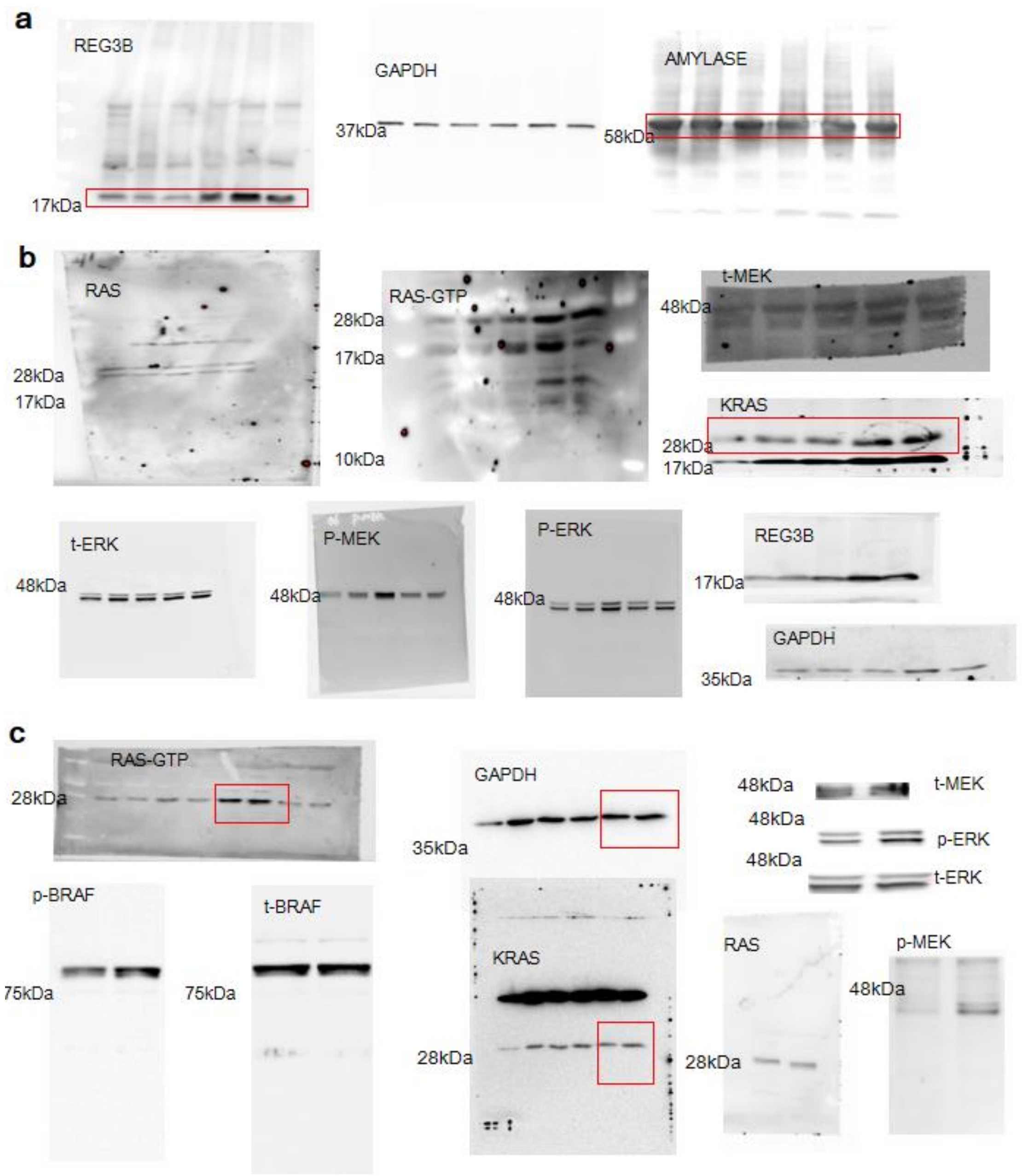

Supplementary Figure 4 – Related to Figure 2, 5a, 5b

a Western blots showing dramatically higher REG3B protein level and significantly lower AMYLASE protein level in the pancreatic tissue of caerulein-treated mice than that of PBS-treated mice. **b** Western blots showing activated RAS-BRAF-MEK-ERK signaling pathway in caerulein plus REG3B protein treated wild-type mice and REG3B overexpressed transgenic mice at Day 7. **c** Western blots showing activated RAS-BRAF-MEK-ERK signaling pathway upon REG3B stimulation in 266-6 for 2 days. Primary antibodies are indicated on top. GAPDH used as loading controls.

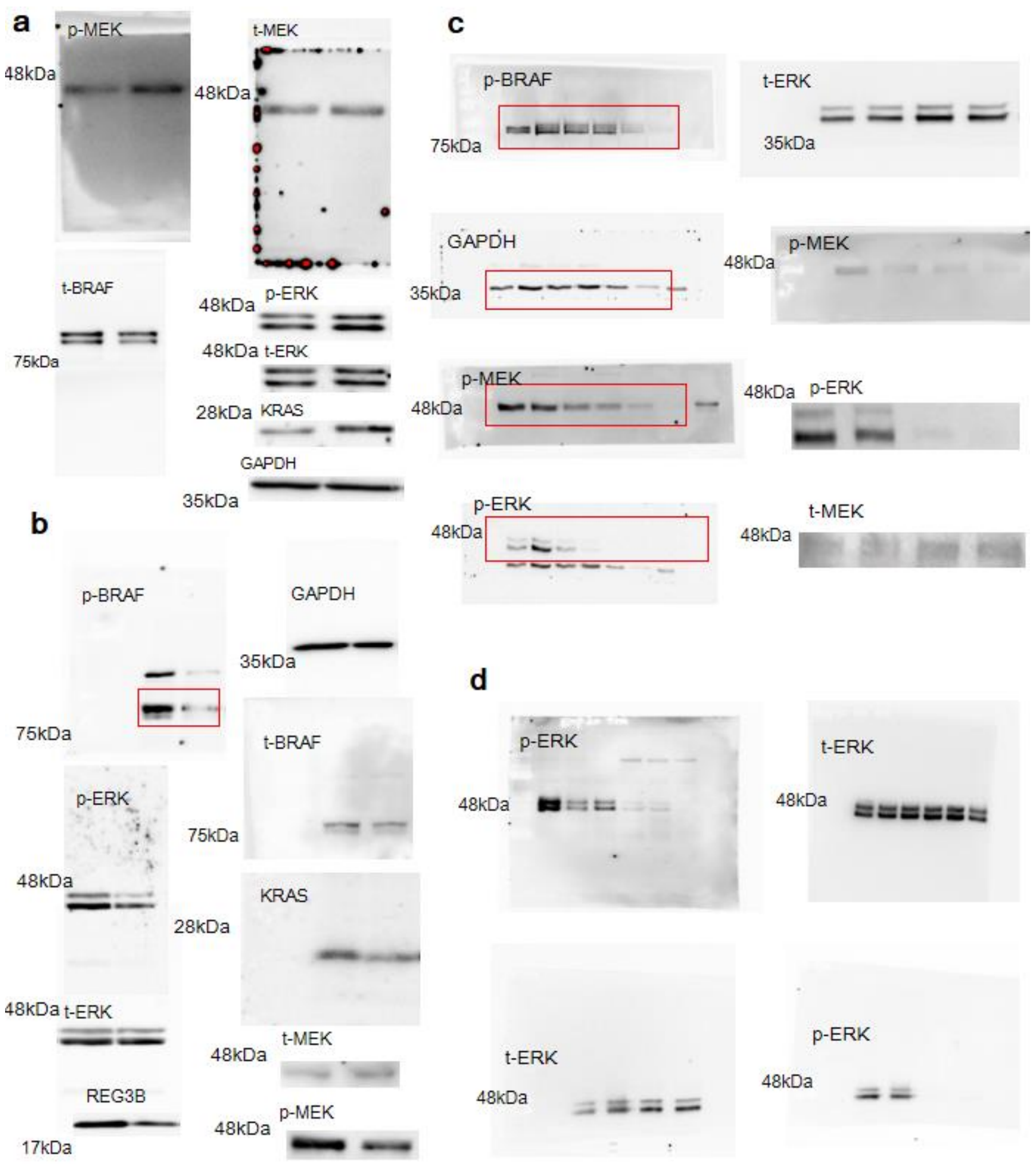

Supplementary Figure 5 – Related to Figure 5b-5e

a Western blots showing activated RAS-BRAF-MEK-ERK signaling pathway upon REG3B stimulation in 266-6 for 2 days. **b** Western blots showing that si-*Reg3b* deactivated RAS-BRAF-MEK-ERK signaling pathway. **c** Western blot showing that BRAF inhibitor, LY3009120 (5μM) decreased p-MEK and p-BRAF in a dose dependent manner and inhibited REG3B-induced BRAF phosphorylation. **d** Western blots showing that MEK inhibitor, Trametinib (100nM), decreased p-ERK in a dose dependent manner and inhibited REG3B-induced ERK phosphorylation. Primary antibodies are indicated on top. GAPDH used as loading controls.

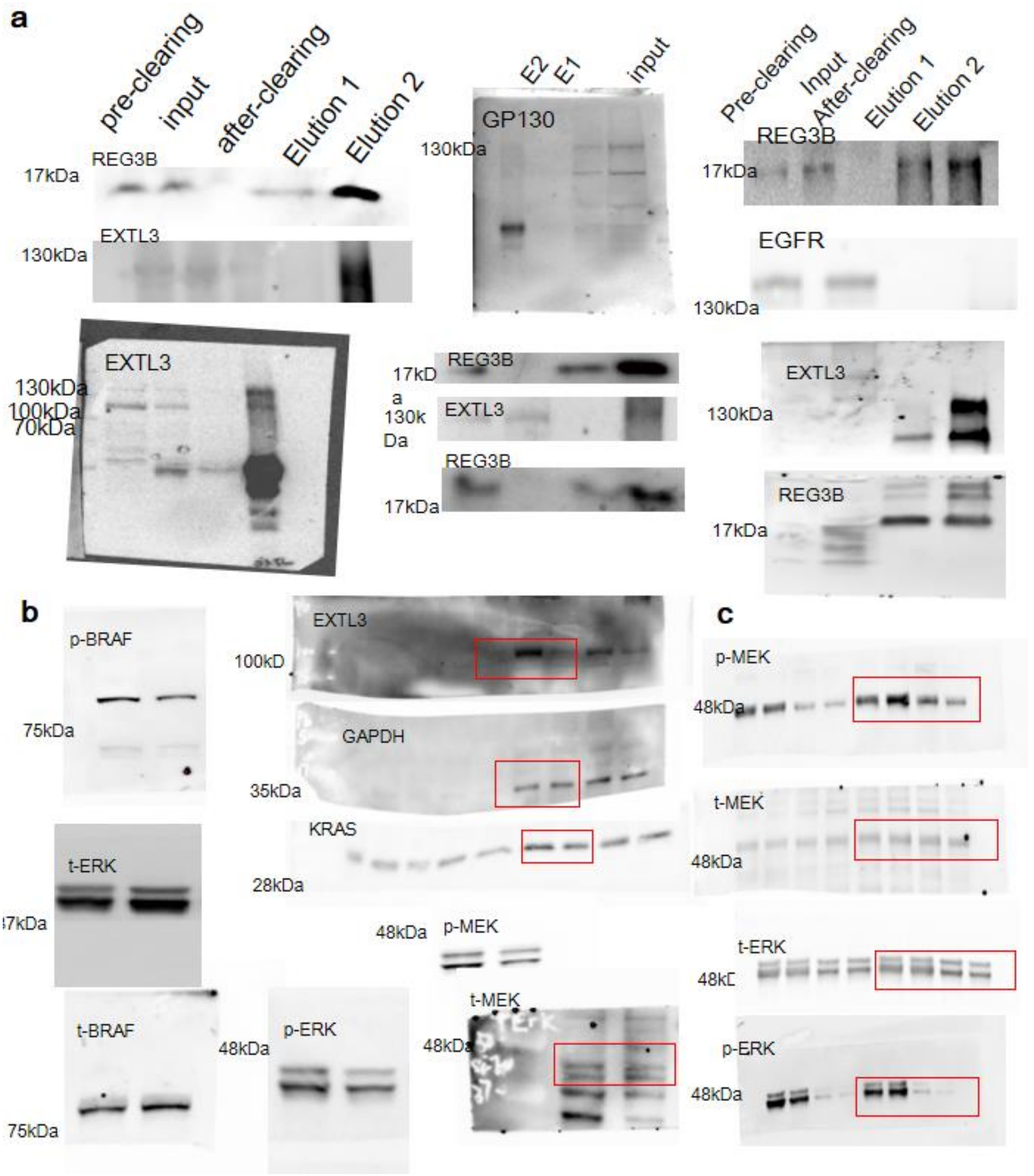

Supplementary Figure 6 – Related to Figure 6b, 6d

a Co-IP images showing interactions between REG3B and EXTL3. **b** Western blots showing inhibition of RAS-RAF-MEK-ERK phosphorylation by si-*Extl3*. **c** Western blots showing that si-*Extl3* dramatically decreased REG3B-induced activation of MEK-ERK signaling pathway. Primary antibodies are indicated on top. GAPDH used as loading controls.

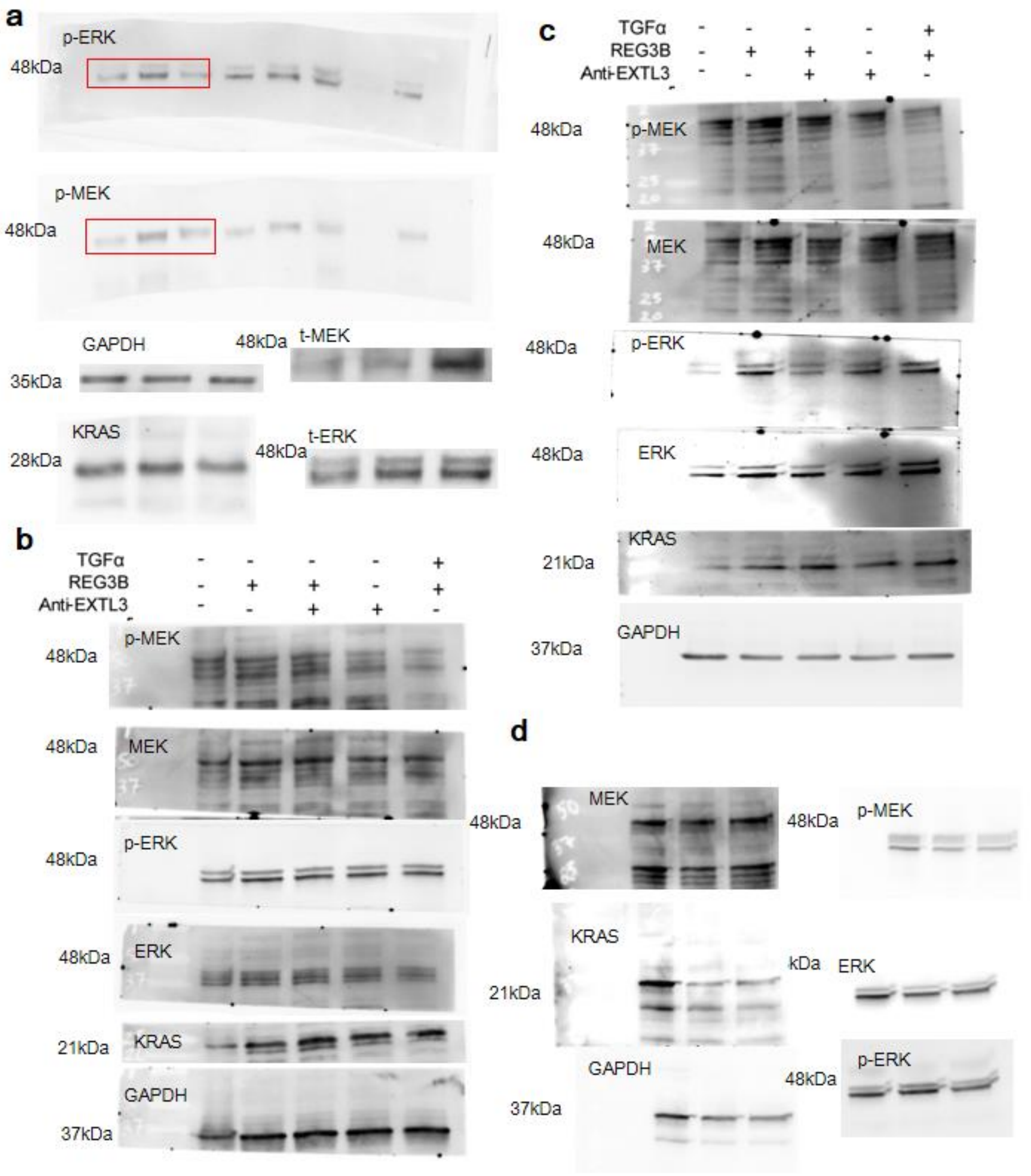

Supplementary Figure 7 – Related to Figure 6f

a Western blots showing that anti-EXTL3 blocked REG3B-induced phosphorylation of MEK-ERK. **b** Western blots showing that EXTL3 neutralizing antibody inhibits REG3B-activated RAS-MEK-ERK signaling pathway in primary mouse acinar cells. **c** Western blots showing that EXTL3 neutralizing antibody inhibits REG3B-activated RAS-MEK-ERK signaling pathway in AR42J cell line. **d** Western blots showing that EXTL3 neutralizing antibody inhibits REG3B-activated RAS-MEK-ERK signaling pathway in primary human acinar cells.

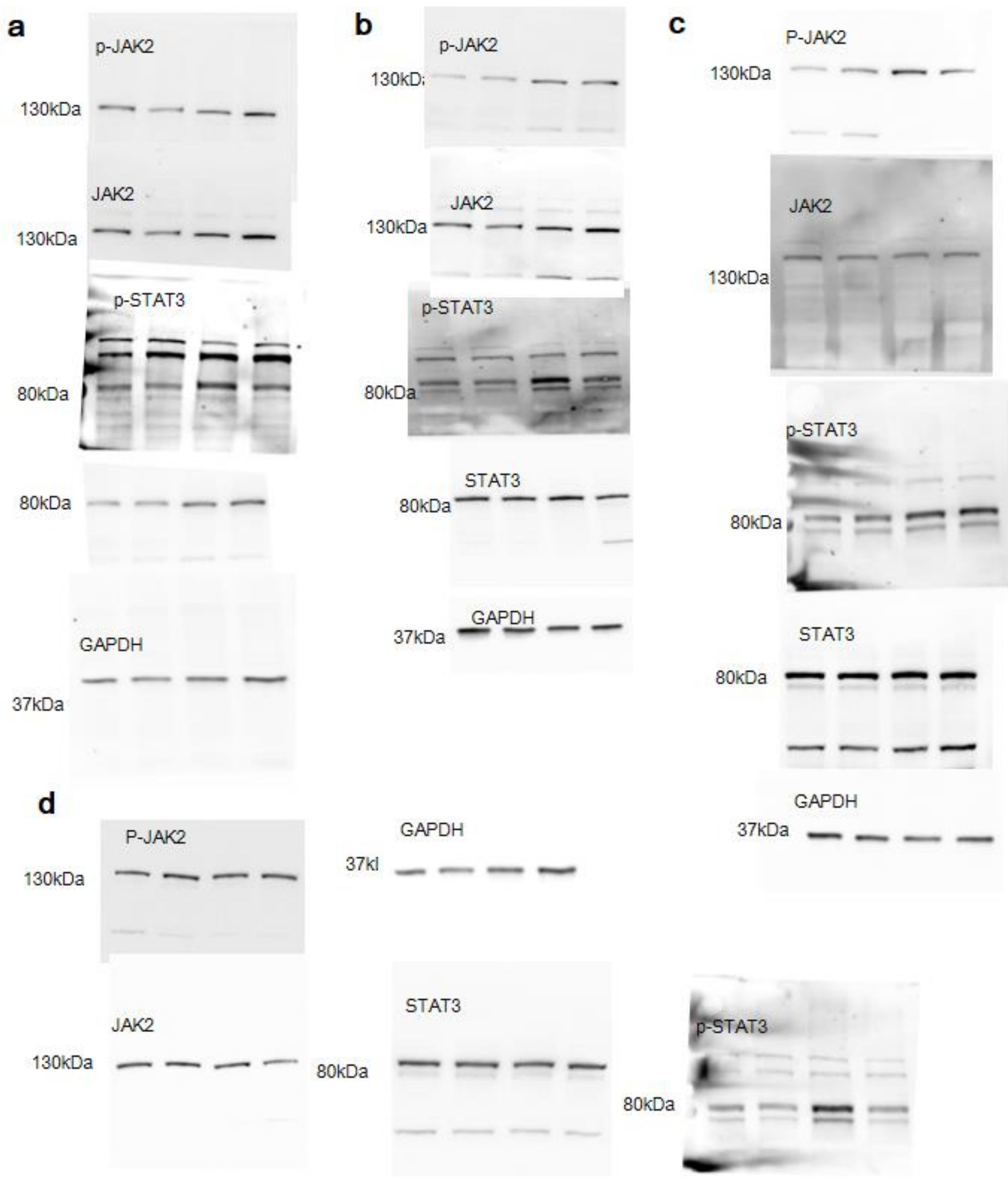

Supplementary Figure 8 – Related to Fig.7

a Western blots showing that neither EXTL3 neutralizing antibody nor REG3B dramatically altered JAK2/STAT3 signaling pathway in 266-6 cell line in 30mins. **b** Western blots showing that REG3B alone failed to activate JAK2/STAT3 signaling pathway in 266-6 cell line for 72 hours. **c** Western blots showing that REG3B alone failed to activate JAK2/STAT3 signaling pathway in AR42J cell line for 30 minutes. **d** estern blots showing that REG3B alone failed to activate JAK2/STAT3 signaling pathway in AR42J cell line for 72 hours.

Supplementary Table 1 Basic information of human pancreas samples used in the current study

Age	Gender	Procedure
43	Male	Whipple resection
31	Female	Distal Pancreatectomy
65	Male	Distal Pancreatectomy
81	Male	Whipple resection

Supplementary Table 2 PCR primers used in the current study

Gene	Primers	Sequence
Mist1	*Mist1-F*	GCTCTCCAAGATCGAGAC
	Mist1-R	GGGAGGCGGCTGCTGGACAT
Amylase	*Amylase-F*	CCTTGGTGGGAAAGATA
	Amylase-R	ACAGCATCCACATAAAT
Pft1a	*Pft1a-F*	CAACGACGCCTTCGAGG
	Pft1a-R	AGCTCGCTGAGGAAGTT
Cpa	*Cpa-F*	GTGGACCCCAACAGGAACTGGG
	CPpa-R	GTGGATGGAGATGAAGGCCTTG
Sox9	*Sox9-F*	GCGTATGAATCTCCTGGACCC
	sox9-R	CCTCGCTCTCCTTCTTCAGAT
Ck19	*Ck19-F*	GAGCTGGCCTACCTGAAGAAG
	Ck19-R	GGCTTCAGCATCCTTCCGGTTC
Nestin	*Nestin-F*	CAGATGTGGGAGCTCAATCG
	Nestin-R	CTCCAGCTCTTCAGCCAGGT
Rpl13a	*Rpl13a-F*	GCGGCTGCCGAAGATGGCG
	Rpl13a-R	ACCACCACCTTCCGGCCCAG
Ywhaz	*Ywhaz-F*	GGCAGCCTGCATGAAGTCTGT
	Ywhaz-R	GATGACCTACGGGCTCCTACAAC
Reg3a	*hReg3a-F*	GGTTACCCTATGTCTGCAAGTTCA
	hReg3a-R	GATGAGTTGCACACCAAACACA

Supplementary Table 3 siRNA oligonucleotides for *Extl3* and *Reg3b*

	Source	Identifier
Extl3 siRNA	OriGene	Cat#SR421199
Scramble si-*Extl3*	OriGene	Cat#SR421199
Reg3b siRNA (Mouse)	OriGene	Cat#SR404149
Scramble si-*Reg3b*(Mouse)	OriGene	Cat#SR404149
