# Supplementary material for: REG3A/REG3B promotes acinar to ductal metaplasia through binding to EXTL3 and activating the RAS-RAF-MEK-ERK signaling pathway
Source: Commun Biol. 2021 Jun 7;4:688. doi: 10.1038/s42003-021-02193-z (PMC8184755; doi:10.1038/s42003-021-02193-z)
Supplement: Supplementary file 3 — Description of Supplementary Files [file 42003_2021_2193_MOESM3_ESM.pdf]

Description of Additional Supplementary Files

File name: Supplementary Data 1

Description: All source data underlying the graphs presented in the main figures.
