# Supplementary material for: REG3A/REG3B promotes acinar to ductal metaplasia through binding to EXTL3 and activating the RAS-RAF-MEK-ERK signaling pathway
Source: Commun Biol. 2021 Jun 7;4:688. doi: 10.1038/s42003-021-02193-z (PMC8184755; doi:10.1038/s42003-021-02193-z)
Supplement: Supplementary file 5 — Reporting Summary [file 42003_2021_2193_MOESM5_ESM.pdf]

nature research

Corresponding author(s): Zu-hua Gao

Last updated by author(s): April 24, 2021

Reporting Summary

Nature Research wishes to improve the reproducibility of the work that we publish. This form provides structure for consistency and transparency in reporting. For further information on Nature Research policies, seeAuthors & Referees and theEditorial Policy Checklist .

Statistics

For all statistical analyses, confirm that the following items are present in the figure legend, table legend, main text, or Methods section.

n/a	Confirmed	
☐	☒	The exact sample size (*n*) for each experimental group/condition, given as a discrete number and unit of measurement
☐	☒	A statement on whether measurements were taken from distinct samples or whether the same sample was measured repeatedly
☐	☒	The statistical test(s) used AND whether they are one- or two-sided<br>*Only common tests should be described solely by name; describe more complex techniques in the Methods section.*
☒	☐	A description of all covariates tested
☐	☒	A description of any assumptions or corrections, such as tests of normality and adjustment for multiple comparisons
☐	☒	A full description of the statistical parameters including central tendency (e.g. means) or other basic estimates (e.g. regression coefficient) AND variation (e.g. standard deviation) or associated estimates of uncertainty (e.g. confidence intervals)
☐	☒	For null hypothesis testing, the test statistic (e.g. *F*, *t*, *r*) with confidence intervals, effect sizes, degrees of freedom and *P* value noted<br>*Give P values as exact values whenever suitable.*
☒	☐	For Bayesian analysis, information on the choice of priors and Markov chain Monte Carlo settings
☒	☐	For hierarchical and complex designs, identification of the appropriate level for tests and full reporting of outcomes
☒	☐	Estimates of effect sizes (e.g. Cohen's *d*, Pearson's *r*), indicating how they were calculated

Our web collection on statistics for biologists contains articles on many of the points above.

Software and code

Policy information about availability of computer code

Data collection	*Provide a description of all commercial, open source and custom code used to collect the data in this study, specifying the version used OR state that no software was used.*
Data analysis	*Provide a description of all commercial, open source and custom code used to analyse the data in this study, specifying the version used OR state that no software was used.*

For manuscripts utilizing custom algorithms or software that are central to the research but not yet described in published literature, software must be made available to editors/reviewers. We strongly encourage code deposition in a community repository (e.g. GitHub). See the Nature Research guidelines for submitting code & software for further information.

Data

Policy information about availability of data

All manuscripts must include a data availability statement. This statement should provide the following information, where applicable:

- Accession codes, unique identifiers, or web links for publicly available datasets
- A list of figures that have associated raw data
- A description of any restrictions on data availability

The datasets generated and/or analyzed during the current study are available from the corresponding author under an reasonable request. There is no restriction on data availability.

Field-specific reporting

Please select the one below that is the best fit for your research. If you are not sure, read the appropriate sections before making your selection.

Life sciences study design

All studies must disclose on these points even when the disclosure is negative.

Sample size	Sample size were provided in the manuscript. The sample size were determined based on literature and standard experimental design. We did not perform sample size calculation.
Data exclusions	No data was excluded
Replication	We were able to duplicate, or even triplicate our results in differential settings: in vitro, in vivo, in human tissue, and in animal models.
Randomization	Sample allocation was random.
Blinding	All data were analyzed in a blinded way.

Reporting for specific materials, systems and methods

We require information from authors about some types of materials, experimental systems and methods used in many studies. Here, indicate whether each material, system or method listed is relevant to your study. If you are not sure if a list item applies to your research, read the appropriate section before selecting a response.

Materials & experimental systems

n/a	Involved in the study	
☐	☒	Antibodies
☒	☐	Eukaryotic cell lines
☒	☐	Palaeontology
☐	☒	Animals and other organisms
☒	☐	Human research participants
☒	☐	Clinical data

Methods

n/a	Involved in the study	
☒	☐	ChIP-seq
☒	☐	Flow cytometry
☒	☐	MRI-based neuroimaging

Antibodies

Antibodies used

CK19 DSHB TROMAIII 1:100 IF
Kras Novus NBP2-45536 1:2000 WB
Amylase CST 3796S 1:1000 WB; 1:100 IF
Reg3A R&D system MAB5965 1:1000 WB; 1:100 IF; 1:150 IHC
mReg3β R&D system AF5110 1:1000 WB; 1:100 IF
rReg3β R&D system AF1996 1:1000 WB; 1:100 IF
p-Erk CST 4370S 1:1000 WB
Erk CST 5013S 1:1001 WB
RAS CST 8821 1:200 WB
GAPDH Thermofisher 1:1000 WB
Tubulin CST 2128S 1:1000 WB
EXTL3 NOVUS NBP1-31645 1:1000 WB
Anti-Mouse Abcam ab97040 1:5000 WB
Anti-Rabbit CST 7074 1:5001 WB
Anti-Goat R&D system HAF109 1:5002 WB
Anti-Sheep R&D system HAF016 1:5003 WB

Validation

Antibodies were validated in mouse, rat and human acinar cells or cell lines.

Animals and other organisms

Policy information about studies involving animals; ARRIVE guidelines recommended for reporting animal research

Laboratory animals	Animal protocols were approved by the McGill University Animal Care Committee (AUP7052). The C57BL/6J (6-12 wks old) mice were purchased from the Jackson Laboratory. RIP-I/Reg3β transgenic mice were established and validated by genotyping, as previously described by our group (xiong, et al Endocrinology and Metabolism 2011; 300:E669-680).
Wild animals	N/A

Field-collected samples	N/A
Ethics oversight	Animal protocols were approved by the McGill University Animal Care Committee (AUP7052).This study was approved by the Institutional Research Ethics Board of McGill University Health Center (MP-37-2018-4399).

Note that full information on the approval of the study protocol must also be provided in the manuscript.
